# Differential Metabolomic Responses of Kentucky Bluegrass Cultivars to Low Nitrogen Stress

**DOI:** 10.3389/fpls.2021.808772

**Published:** 2022-01-28

**Authors:** Xiaoyang Sun, Zhixin Guo, Yiwei Jiang, Ligang Qin, Zhenjie Shi, Lili Dong, Liangbing Xiong, Runli Yuan, Wenjing Deng, Hanfu Wu, Qingqing Liu, Fuchun Xie, Yajun Chen

**Affiliations:** ^1^College of Animal Science and Technology, Northeast Agricultural University, Harbin, China; ^2^College of Horticulture, Northeast Agricultural University, Harbin, China; ^3^Department of Agronomy, Purdue University, West Lafayette, IN, United States; ^4^College of Horticulture, Nanjing Agricultural University, Nanjing, China

**Keywords:** carbon–nitrogen metabolism, low nitrogen, Kentucky bluegrass, metabolomics, physiology

## Abstract

Kentucky bluegrass (*Poa pratensis* L.) is a cool-season turfgrass species that responds strongly to nitrogen (N), but the metabolomic responses of this grass species to N supply is unknown. The N-tolerant cultivar Bluemoon and N-sensitive cultivar Balin were exposed to normal N (15 mM) and low N (0.5 mM) for 21 days for identification of differentially expressed metabolites (DEMs) between normal N and low N treatments. Balin had more reductions of chlorophyll and total soluble protein concentrations and a higher accumulation of superoxide radicals under low N stress. A total of 99 known DEMs were identified in either cultivar or both including 22 amino acids and derivatives, 16 carbohydrates, 29 organic acids, and 32 other metabolites. In Bluemoon, β-alanine metabolism was most enriched, followed by alanine, aspartate, and glutamate metabolism, biosynthesis of valine, leucine, and isoleucine biosynthesis, and glycine, serine, and threonine metabolism. In Balin, alanine, aspartate, and glutamate metabolism were most enriched, followed by the tricarboxylic acid (TCA), glyoxylate and decarbohydrate metabolism, and carbon fixation. Bluemoon generally maintained higher TCA cycle capacity and had more downregulated amino acids, while changes in more organic acids occurred in Balin under low N stress. Some metabolite changes by low-N stress were cultivar-specific. The results suggested that regulation of metabolites related to energy production or energy saving could contribute to low N tolerance in Kentucky bluegrass.

## Introduction

Kentucky bluegrass (*Poa pratensis* L.) is a popular cool-season perennial turf and forage grass with attractive leaf and plant shapes. This species has a rhizomatous growth habit, which can form populations by vegetative propagation of tiller nodes and rhizomes (Chen et al., 2019; Saud et al., 2020). Nitrogen (N) is one of the key nutrients affecting the growth and development of grass plants. A proper amount of N application can promote vegetative reproduction, improve turf quality, and increase stress resistance of perennial grass plants (Saud et al., 2017; Gao S. et al., 2020). However, when N supply is insufficient, plants often show leaf chlorosis, stunted growth, and reduced biomass. Total forage dry matter yield decreased with decreases in N rate in Kentucky bluegrass and other perennial grass species (Zemenchik and Albrecht, 2002). The supply of N below a certain level resulted in very poor color, shoot growth and yield of Kentucky bluegrass (Nelson, 1984). Moreover, low N stress decreased photosynthetic rate, chlorophyll content, chlorophyll fluorescence, and the non-photochemical quenching, especially in the N-sensitive cultivar of Kentucky bluegrass (Sun et al., 2021). Reductions in plant height, shoot fresh and dry weight and shoot carbon content was also observed in the N-sensitive accession of perennial ryegrass (*Lolium perenne* L.) (Yao et al., 2019). Thus, a better understanding of plant responses to N supply especially under low N condition is crucial for fertility management programs of perennial grasses including Kentucky bluegrass.

Plant responses to external N supply often result in many cellular and molecular changes related to N metabolism. For example, low N stress reduced leaf activities of glutamine synthetase and glutamate synthetase in Kentucky bluegrass, but lower activity of nitrate reductase was found only in the sensitive cultivar exposed to low N condition (Sun et al., 2021). Furthermore, the identified genes involved in carbon metabolism were highly expressed in the tolerant cultivar (Sun et al., 2021). Low N stress can alter not only gene expression and protein synthesis but also the level of metabolites (Kusano et al., 2011). The primary metabolites (e.g., sugars, organic acids, and amino acids) and secondary metabolites (e.g., flavonoids) are the catalytic products of enzymatic reactions and may serve as active regulators in complex metabolic pathways and networks including carbon and N homeostasis. Therefore, changes of metabolites to low N availability reflect the capacity of plant tolerance to low N stress. In rice (*Oryza sativa* L.), high N availability inhibited N assimilation and aromatic metabolism pathways, while tricarboxylic acid (TCA) was enhanced under low N conditions to promote N transport and assimilation (Xin et al., 2019). Enrichment of metabolites such as pyroglutamate, glutamate, 2-oxoglutarate, sorbose, glycerate-2-P, and phosphoenolpyruvic acid were found in a backcross introgression line of rice relative to its recurrent parent, indicating that these metabolites could be involved in low-N stress tolerance (Zhao et al., 2018). Moreover, the TCA cycle, shikimic acid pathway, synthetase/glutamate synthase (GS/GOGAT) cycle, and accumulation of most organic acids were enhanced in the low N-tolerant genotype of wild soybean (*Glycine soja* Sieb.) but reduced in the common genotype (Zhao et al., 2020). In wild barley (*Hordeum spontaneum* L.), low N affected the glycolysis, TCA, and pentose phosphate pathways, while the tolerant genotype accumulated energy-saving amino acids, distributed carbon to roots, and had a higher capacity to maintain redox homeostasis (Quan et al., 2016). Metabolic changes under low N stress also depend on the growth stage and age of the plants. It was reported that key amino acids and non-protein N metabolism (e.g., aspartic acid and proline) was highly accumulated in old leaves of wild soybean relative to cultivated soybean (*Glycine max* L.), which contributed to higher survival and growth of young leaves in wild soybean under N deficiency (Liu et al., 2019). A study in wheat (*Triticum aestivum* L.) indicated that flavonoids and their related derivatives might be biomarkers of low-N stress conditions (Zhang et al., 2017). Collectively, metabolites are important cellular components and play crucial roles in low N tolerance of plants.

The study of metabolic profiling has increased our understanding of plant systematic regulatory mechanisms, especially for gaining insights on metabolite alterations in plants exposed to environmental stress. Although metabolites and related pathways have been characterized in different plant species to low N stress including grain crops (Heyneke et al., 2017; Zhao et al., 2018; Sheflin et al., 2019; Xin et al., 2019; Ganie et al., 2020), vegetables (Sung et al., 2015; Gao H. et al., 2020; Zhou et al., 2021), perennial grass (He et al., 2021), tree (Song et al., 2020), and algal microorganisms (Vello et al., 2018), the results were inconsistent in different plant species. For example, low N availability decreased the levels of N-containing metabolites, organic acids and amino acids and increased soluble sugar content in maize (*Zea mays* L.) lines with contrasting N responses (Ganie et al., 2020), but the tolerant genotype accumulated more amino acids and organic acids in wild barley and soybean when exposed to low N stress (Quan et al., 2016; Zhao et al., 2020). It has been suggested that a challenge is posed to identify genotype-specific metabolic markers in wheat due to potential factors related to metabolome homeostasis and genetic diversity (Heyneke et al., 2017). Owing to the complex metabolic pathways and genotype by environment interaction, responses of plant metabolism to low N stress could be influenced by various factors, such as stress duration and intensity, type of tissue, and stage of plant growth and development, etc. Therefore, our current knowledge on metabolomic responses of plants to variable N supply is still lacking, especially for perennial grasses.

In this study, we explored and compared the expression of metabolites in two Kentucky bluegrass cultivars with contrasting N responses aimed at identifying differentially expressed metabolites (DEMs) under normal and low N treatments. We hypothesized that changes of DEMs contributed to differential responses of Kentucky bluegrass cultivars to low N supply. These results would help elucidate the role of metabolites in low N tolerance of Kentucky bluegrass. Furthermore, the identified metabolites could potentially be used for developing cultivars with improved low N tolerance and N-use efficiency in perennial grass species.

## Materials and Methods

### Plant Materials, Growing Conditions, and Experimental Design

Two Kentucky bluegrass cultivars (Bluemoon and Balin) were collected from field plots at the Horticulture Experimental Station of Northeast Agricultural University (Harbin, China; 128°04′ E, 44°50′ N) and were used for the experiment. Bluemoon was more tolerant to low N stress than Balin (Sun et al., 2021). Two-year-old turf plants were transplanted to pots (15 cm in diameter and 45 cm in height) filled with a mixture of sand and vermiculite (2:1) in a greenhouse. The plants were grown in the greenhouse for 30 days, with average day/night temperatures of 22/15°C and an average 600 μmol m^–2^ s^–1^ photosynthetically active radiation for 12 h. During the growth period, plants were cut once a week to 6 cm, watered every 2 days, and fertilized once a week with a 60 mL Hoagland solution. Three weeks before the application of N treatments, all pots were transferred to a growth chamber, with temperatures of 25/15°C (day/night) and 65% relative humidity. Two weeks after adaptation to the growth chamber environment, the pots were rinsed with deionized water for 1 week and followed by two N treatments: low N (LN, 0.5 mM NO_3_^–^) and normal N (NN, 15 mM NO_3_^–^) with Hoagland solution used as the N source. The N rates were chosen based on our previous study that two cultivars differed in their responses to these two N treatments (Sun et al., 2021). The experiment was a completely randomized design with 2 × 2 factorial arrangements (two N treatments, two cultivars). After 21 days of N treatments, the leaves were collected for metabolic profiling. Six samples were used for metabolomics analysis for both cultivars under LN and NN, respectively.

### Physiological Measurements

Chlorophyll *a* and *b* were determined according to the method of Lichtenthaler (1987). Approximately 0.2 g of fully expanded fresh leaves were ground and extracted with ethanol (95%, v/v) in a dark room. After filtering, ethanol was added to a final volume of 25 mL. The absorbance was read at 665 and 649 nm using a UV-Vis spectrophotometer (T6 New Century, Beijing, China). The superoxide anion (O_2_^•–^) was measured by the method of Tian et al. (2003). Briefly, fresh leaf sample (0.5 g) was homogenized in an ice-cold mortar with 2 mL of 50 mM phosphate buffer (PBS) (pH 7.8). The homogenate was centrifuged at 12,000 × *g* for 15 min at 4°C, and then the supernatant was collected. The 1 mL extract was mixed with 1 mL of 10 mM hydroxylamine hydrochloride, incubated in a water bath (25°C) for 20 min, and then the absorbance was read at 530 nm. The malondialdehyde (MDA) content was determined according to the method described by Dhindsa et al. (1981) with modifications. Fresh leaf sample (0.2 g) was ground and homogenized with 10% trichloroacetic acid (w/v). The homogenates were centrifuged at 4000 × *g* for 10 min and the supernatant was collected. The 1 mL of supernatant was mixed with 5 mL of 20% trichloroacetic acid (w/v) containing 0.6% thiobarbituric acid (w/v). The mixture was maintained in a boiling water bath for 15 min and then quickly cooled in ice, and the mixture was centrifuged at 4000 × *g* for 10 min. The absorbance was read at 532 and 600 nm. The concentration of MDA was calculated using MDA’s extinction coefficient of 155 mM^–1^ cm^–1^ (Heath and Packer, 1968). For the total soluble protein (TSP) measurement, approximately 0.2 g leaves were mixed with 5 mL of extraction buffer (0.05 M Tris–HCl, pH 8.0, containing 2 mM Mg^2+^, 2 mM DTT, and 0.4 M sucrose). The mixture was homogenated in an ice bath, and then centrifuged at 12,000*g* at 4°C for 20 min. A 0.5 mL aliquot of the crude extraction sample was diluted to 10 mL with deionized water, and 2 mL of Coomassie Brilliant Blue G-250 was added. The absorbance was read at 595 nm (Bradford, 1976).

### Extraction, Detection, and Analysis of Metabolites

Approximately 50 mg of the leaf samples was put into a 2 mL microcentrifuge tube and mixed with 480 μL of extraction solution (methanol: chloroform = 3:1) (Schenck et al., 2020). Ten microliter of L-2-chlorophenylalanine was then added as an internal standard and vortexed for 30 s. The solution was homogenized in a ball mill at a frequency of 45 Hz for 4 min and subjected to ultrasonic treatment for 5 min, and then centrifuged for 15 min at 12,000 rpm at 4°C. Approximately 350 μL of supernatant was transferred to a 1.5 mL microcentrifuge tube, and the sample was dried completely in a vacuum concentrator. Subsequently, the sample was mixed with 80 μL of methoxyamine hydrochloride (20 mg mL^–1^ in pyridine) and incubated at 80°C for 30 min. All samples were analyzed by a combination of a gas chromatography system (United States, TRACE GC 1300) and a mass spectrometer (GC-TOF-MS) (Girardello et al., 2020). LECO’s Chroma TOF 4.3 × software and LECO Fiehn Rtx5 database were used for original peak extraction, data baseline filtering, baseline calibration, peak alignment, deconvolution analysis, peak identification, and peak area integration (Chang et al., 2020). The retention time index (RI) method was used for peak identification, and the RI tolerance was set at 5000. The metabolites were identified using a database developed by FiehnLib (Kind et al., 2009).

### Identification of Differentially Expressed Metabolites

Relevant parameter data was input into the SIMCA 14.1 software package for principal component analysis (PCA) and latent structure discrimination analysis (Orthogonal Partial Least Squares Discrimination Analysis, OPLS-DA) (Sun et al., 2021). Using OPLS-DA model, the first principal component in the projection importance (VIP) value (VIP > 1) was combined with a t test (*P* < 0.05) to determine the DEMs in the paired comparison as follows: Bluemoon under low N treatment (LLN) vs normal N (LNN); Balin under low N (BLN) vs normal N (BNN).

### Data Analysis

Data were analyzed using one-way analyses of variance with SPSS v10.0 software (SPSS, Inc., Chicago, IL, United States). Mean values were compared with the least significant difference test at the 0.05 probability level. Figures were plotted using R v3.4.0 and GraphPad Prism v 9.00 (Graphpad Company, United States).

## Results

### Physiological Responses to Low N Stress

**FIGURE 1:**
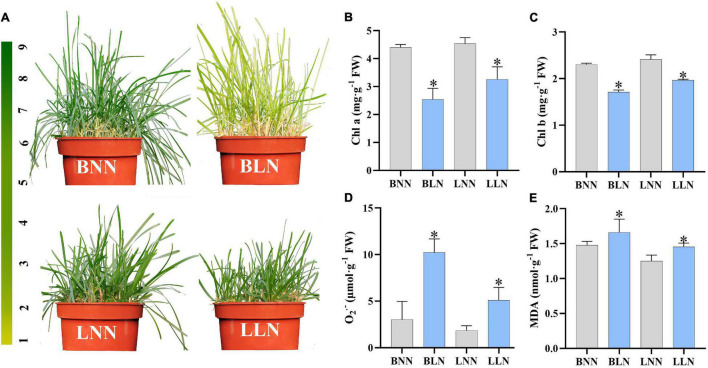
The morphology **(A)**, chlorophyll *a* **(B)**, chlorophyll *b* **(C)**, superoxide anion (O_2_^•–^) **(D)**, and malondialdehyde (MDA) **(E)** under normal nitrogen (NN) and low nitrogen (LN) treatments. BNN, Balin under NN; BLN, Balin under LN; LNN, Bluemoon under NN; LLN, Bluemoon under LN. Asterisk represents significance at *P* < 0.05 between NN and LN for a given cultivar. The bars represented standard deviation.

Leaf color looked green in Balin and Bluemoon under NN, but discoloration appeared in the sensitive cultivar Balin much greater than the tolerant cultivar Bluemoon under LN treatment (Figure 1A). Compared to NN, the contents of Chl *a* and Chl *b* decreased by 42 and 26% for Balin and by 28 and 19% for Bluemoon under LN, respectively (Figures 1B,C). The content of O_2_^•–^ increased by 238% for Balin and 175% for Bluemoon under LN, while MDA content increased by 12% for Balin and 16% for Bluemoon, compared to their NN treatments (Figures 1D,E).

### The Identified Differentially Expressed Metabolites and Pathway Enrichment

**FIGURE 2:**
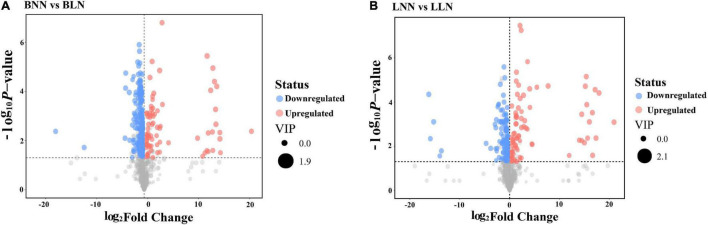
Volcano map analysis of differentially expressed metabolites (DEMs) between normal nitrogen (NN) and low nitrogen (LN) treatments. The red, blue, and gray circles represent the DEMs that were upregulated, downregulated, and no significant changes, respectively. BNN, Balin under NN; BLN, Balin under LN; LNN, Bluemoon under NN; LLN, Bluemoon under LN. **(A)** BNN vs BLN; **(B)** LNN vs LLN.

**FIGURE 3:**
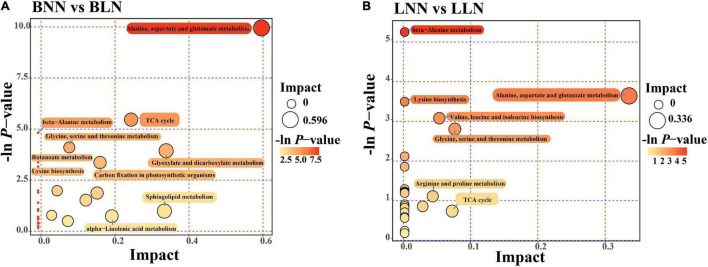
Differentially expressed metabolites (DEMs) KEGG pathway enrichment analysis for comparison between normal nitrogen (NN) and low nitrogen (LN) treatments. The abscissa pathway impact represents the influencing factor of the path topological analysis, and the ordinate –log(*P*) represents the *P*-value of the pathway enrichment analysis. The circle size indicates the influence factor of topological analysis and color of the circle indicates the *P*-value of the enrichment analysis. BNN, Balin under NN; BLN, Balin under LN; LNN, Bluemoon under NN; LLN, Bluemoon under LN. **(A)** BNN vs BLN; **(B)** LNN vs LLN.

After screening, Balin had 186 upregulated DEMs and 80 downregulated DEMs, while Bluemoon had 116 upregulated and 82 downregulated DEMs in response to LN, compared to the NN treatment (Figures 2A,B and Supplementary Tables 1, 2). Of these, there were 99 known DEMs identified in either cultivar or both. In Balin, the metabolic pathways related to N metabolism such as alanine, aspartate, and glutamate metabolism were most significantly enriched followed by the TCA cycle, glyoxylate and decarbohydrate metabolism, and carbon fixation, etc. (Figure 3A). In Bluemoon, the β-alanine metabolism pathway showed the most significant enrichment followed by alanine, aspartate, and glutamate metabolism, valine, leucine, and isoleucine biosynthesis, and glycine, serine, and threonine metabolism, etc. (Figure 3B).

### Heatmap Analysis of Differentially Expressed Metabolites

**FIGURE 4:**
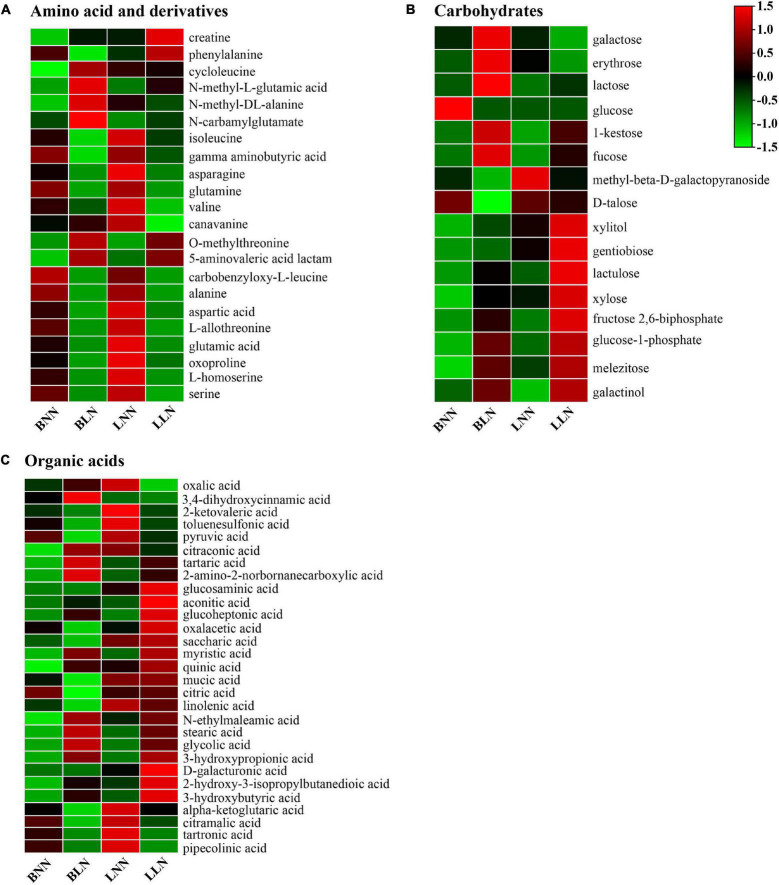
Hierarchical cluster analysis of metabolites under normal nitrogen (NN) and low nitrogen (LN) treatments. Heatmap analysis for amino acid **(A)**, sugars **(B)**, and organic acid **(C)**. Hierarchical clustering was performed on the normalized relative abundance (indicated by color) for DEMs at each concentration point for *n* = 6 samples. BNN, Balin under NN; BLN, Balin under LN; LNN, Bluemoon under NN; LLN, Bluemoon under LN.

There were 99 known DEMs between the LN and NN treatments identified in either cultivar or both, including 22 amino acids and derivatives, 16 carbohydrates, 29 organic acids, and 32 other metabolites (Figure 4 and Supplementary Tables 1, 2). Specifically, among the 22 amino acids and derivatives, 6 were upregulated and 11 were downregulated in Balin and 3 were upregulated and 12 were downregulated in Bluemoon (Figure 4 and Supplementary Tables 1, 2). Of these, 10 amino acids and derivatives showed the same patterns in both cultivars, with two upregulated and eight downregulated between the LN and NN treatments. Moreover, significant changes in some amino acids and derivatives were observed only in one cultivar but not in the other. The levels of cycloleucine, *N*-methyl-L-glutamic acid, *N*-methyl-DL-alanine, and *N*-carbamylglutamate were upregulated and phenylalanine, glutamine, and γ-aminobutyric acid (GABA) were downregulated only in Balin, while the upregulated creatine and downregulated isoleucine, asparagine, valine, and canavanine were noted only in Bluemoon (Figure 4A and Supplementary Tables 1, 2).

Among the identified carbohydrates between the LN and NN treatments, eight were upregulated and three were downregulated in Balin and five were upregulated and two were downregulated in Bluemoon (Figure 4B and Supplementary Tables 1, 2). Of these, melezitose and galactinol were upregulated in both cultivars. However, the upregulated expression of lactose, 1-kestose, fucose, xylitol, xylose, and glucose-1-phosphate, and downregulated glucose, D-talose and methyl-β-D-galactopyranoside were found only in Balin, while the upregulated level of gentiobiose, lactulose, and fructose 2,6-biphosphate and downregulated galactose and erythrose were found only in Bluemoon (Figure 4B and Supplementary Tables 1, 2).

There were more DEMs for organic acids found in Balin, relative to Bluemoon. Specifically, 12 were upregulated and 11 were downregulated in Balin and 7 were upregulated and 9 were downregulated in Bluemoon (Figure 4C and Supplementary Tables 1, 2). Of these, nine organic acids exhibited the same patterns in both cultivars with five upregulated and four downregulated between the NN and LN treatments. The upregulated expression of 3,4-dihydroxycinnamic acid, tartaric acid, 2-amino-2-norbornanecarboxylic acid, glucoheptonic acid, myristic acid, quinic acid, and N-ethylmaleamic acid, and downregulated oxalacetic acid, pyruvic acid, citric acid, linolenic acid, mucic acid, and saccharic acid were found only in Balin (Figure 4C and Supplementary Table 1). The upregulation of glucoheptonic acid and aconitic acid and downregulation of oxalic acid, 2-ketovaleric acid, citraconic acid, and toluenesulfonic acid were shown only in Bluemoon (Figure 4C and Supplementary Table 2).

In another category of DEMs, 11 were shown only in Balin, 10 only in Bluemoon, and 11 were found in both cultivars when comparing NN with the LN treatment (Supplementary Tables 1, 2). Notably, the expressions of atropine (an alkaloid) and hesperitin (a flavonoid) were upregulated only in Balin, not in Bluemoon, while 5-methoxytryptamine (related to melatonin) and guanosine (a nucleic acid component and an organonitrogen compound) were upregulated only in Bluemoon, not in Balin (Supplementary Tables 1, 2). The expressions of cholecalciferol (vitamin D3) and spermidine (a polyamine) were upregulated in both cultivars (Supplementary Tables 1, 2).

### Total Soluble Protein Content and Key Metabolite Ratio

**FIGURE 5:**
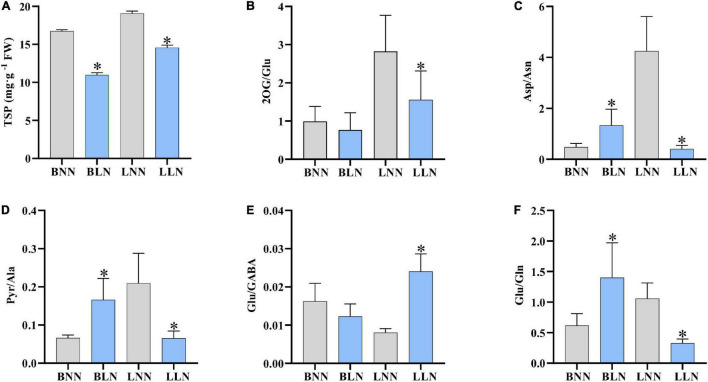
Total soluble proteins **(A)** and metabolic ratios **(B–F)** under normal nitrogen (NN) and low nitrogen (LN) treatments. BNN, Balin under NN; BLN, Balin under LN; LNN, Bluemoon under NN; LLN, Bluemoon under LN. Asterisk represents significance at *P* < 0.05 between NN and LN for a given cultivar. The bars represented standard deviation.

The TSP content decreased by 34.5% for Balin and 23.6% for Bluemoon under LN, compared to the NN treatment (Figure 5A). The ratio of nitrogenous metabolites to their deaminated/deamidated counterparts is shown in Figures 5B–F. The ratios were not consistent in the two cultivars under LN compared to their NN treatments. The 2OG/Glu was unchanged in Balin but decreased in Bluemoon (Figure 5B). Asp/Asn increased in Balin but decreased in Bluemoon (Figure 5C). Pyr/Ala increased in Balin but did not change in Bluemoon (Figure 5D). Glu/GABA and Glu/Gln did not alter in Balin, while increases in Glu/GABA and decreases in Glu/Gln were noted in Bluemoon (Figures 5E,F).

## Discussion

Nitrogen application has a significant impact on leaf color and turf quality. Low N availability accelerates leaf senescence and decreases Chl content (Jiang et al., 2016). In this study, Chl *a*, Chl *b*, and TSP were reduced in both cultivars under low N stress, but to a larger extent in the sensitive Balin (Figures 1B,C, 5A). Our results were consistent with that found in perennial ryegrass (*L. perenne* L.) accessions to low N stress (Jiang et al., 2016; Yao et al., 2019). Meanwhile, the relatively higher amount of O_2_^•–^ observed in Balin suggested a higher degree of oxidative injury occurring to this cultivar, although both cultivars showed enhanced levels of O_2_^•–^ and MDA (Figures 1D,E). Nitrogen deficiency-induced lipid peroxidation and ROS accumulation have been observed in other plant species (Kováčik and Bačkor, 2007; Zakari et al., 2020). The increased oxidative injury might at least partially contribute to declines in Chl and TSP, especially for the sensitive Balin in this study.

Low N stress caused substantial changes in metabolites in both the Balin and Bluemoon cultivars. Alterations of metabolites involved in carbon fixation and the glyoxylate and dicarboxylate metabolism pathways were more significant in Balin (Figures 3A,B). Notably, the glucose level was lower in Balin but not in Bluemoon under LN. In wild barley, the downregulation of glucose was much greater in the sensitive genotype by the end of the low N treatment compared to a previous day of treatment (Quan et al., 2016). The results indicated a lower level of glycolysis potentially occurring in the N sensitive cultivar including Kentucky bluegrass. Since glycolysis is the metabolic pathway that converts glucose into pyruvic acid, a lower glycolysis level could lead to limited energy yields due to a restricted supply for the respiratory substrate, which inevitably influences plant growth (Plaxton, 1996). Therefore, the sensitive Balin might suffer from an energy crisis under an extended period of LN. This was supported by more decline in Chl found in this study and lower physiological activities observed in Balin than in Bluemoon under LN (Sun et al., 2021). However, the changes of particular metabolites related to glycolysis pathway were not consistent in these two cultivars in response to LN. For example, fructose-2, 6-bisphosphate, the most potent stimulator of a key enzyme (6-phosphofructo-1-kinase) of glycolysis, was upregulated in Bluemoon, while upregulation of glucose-1-phosphate was found in Balin. Similarly, the trends of metabolites involved in glycolysis such as glucose-6-phosphate and fructose-6-phosphate was different between the wild and cultivated soybean leaves (Liu et al., 2019). As the last metabolite in the glycolysis pathway, pyruvate plays a critical role in connecting glycolysis with the TCA cycle. However, because of its lower level in Bluemoon and stability in Balin, it seemed that the alteration of pyruvate was uncorrelated with LN tolerance in these two cultivars. At gene expression levels, differentially expressed genes (DEGs) involved in the pyruvate metabolism pathway were significantly enriched in Bluemoon, but not in Balin between the low N and normal N treatments (Sun et al., 2021). The results demonstrated some connections between expressions of genes and metabolites in these two cultivars in response to N supply. In addition, our results of pyruvate alterations were similar to those found in barley that pyruvate significantly decreased under N-starvation conditions (Fataftah et al., 2018). By contrast, increased pyruvate accumulation was observed in soybean, but to greater extent in the low N-tolerant wild genotype than in the common genotype (Zhao et al., 2020). The inconsistency of the results suggested a complex synthesis and regulation of metabolites in the glycolysis pathway influenced by low N stress in different plant species under variable N supplies.

The TCA cycle is an essential pathway toward energy production and N incorporation by providing carbon skeletons. Comparing LN with NN treatment, DEGs involved in the TCA cycle were slightly enriched in Balin, but not in Bluemoon (Sun et al., 2021). These results were generally consistent with the enrichment pattern in metabolites of the TCA cycle in both cultivars in response to N supply (Figure 3). Several key organic acids of the TCA cycle were differentially expressed in these two cultivars under LN stress (Figure 4C). For example, citric acid was reduced in Balin under LN, which was in agreement with previous studies in other plant species (Urbanczyk-Wochniak and Fernie, 2005; Sung et al., 2015). Since citric acid is an upstream metabolite in TCA, a lower level of citric acid may influence the cycle of other metabolites. Citric acid was stable in Bluemoon under LN, which could contribute to TCA levels and overall plant performance. The unchanged citric acid was also noted in different genotypes of soybean with contrasting N responses (Zhao et al., 2020). The upregulation of aconitic acid and downregulation of oxalic acid was found only in Bluemoon under LN, suggesting an inconsistent pattern of some metabolites involved in the TCA cycle. Nevertheless, the enhanced production of aconitic acid might promote the TCA cycle in Bluemoon exposed to LN. When wheat plants were exposed to drought stress, the aconitic acid level decreased, especially in the sensitive cultivar; however, the oxalic acid level was substantially increased in the tolerant cultivar (Guo et al., 2018). The results indicate a role of these organic acids in plant stress tolerance. The other key TCA intermediates, α-ketoglutarate (2-OG) and oxaloacetate (OAA), are precursor metabolites for the biosynthesis of amino acids and critical for carbon/nitrogen metabolism. The trends of two metabolites also differed in two cultivars under LN, with downregulation of 2-OG shown in Bluemoon and downregulation of OAA in Balin. The lower level of 2-OG or OAA could lead to decreases in some amino acids found in this study and in other reports (Sung et al., 2015), which may affect low N tolerance in the plants.

Organic acids are essential to cellular metabolism, which plays an important role in plant stress tolerance (Panchal et al., 2021). Other differentially expressed organic acids could also affect N responses of plants. It was reported that linolenic acid was positively correlated with the shoot dry weight and chlorophyll content of wheat under N-deficient condition (Liu X. et al., 2020). The downregulation of linolenic acid in Balin along with its greater decline in chlorophyll supported this observation. Mucic acid (known as galactaric acid) and saccharic acid can serve as osmoregulators and myristic acid is an antioxidant compound. Downregulation of mucic acid and saccharic acid in Balin may interfere with osmoregulation of plants under LN, while upregulation of myristic acid might indirectly indicate an increased oxidative stress occurring in Balin consistent with its enhanced superoxide content (Figure 1D). There was evidence to support that accumulations of myristic acid and mucic acid increased salinity tolerance of date (*Phoenix dactylifera* L.) following silicon treatments (Jana et al., 2019). The drought tolerant genotype of chickpea (*Cicer arietinum* L.) accumulated saccharic acid and other compounds for osmotic adjustment (Khan et al., 2019). In addition, introgression forms of annual ryegrass (*Lolium multiflorum* Lam.)/tall fescue (*Festuca arundinacea* Schreb.) with the capacity for drought recovery contained higher amounts of compounds involved in osmoprotection including galactaric acid (Perlikowski et al., 2016). Collectively, higher levels of some specific organic acids could contribute to plant stress tolerance including low N stress.

Amino acid metabolism is of crucial importance in N metabolism, signaling processes, and plant stress response (Hildebrandt et al., 2015). Nitrogen deficiency decreased accumulation of amino acids in plants (Fritz et al., 2006; Krapp et al., 2011; Amiour et al., 2012; Sung et al., 2015; Liu D. et al., 2020). Our results agreed with those observations, as most of the differentially expressed amino acids and derivatives were downregulated in either cultivar or both (Figure 4A). The results indicated that low N stress inhibited amino acid metabolism. On the other hand, since amino acid synthesis is energy consuming, reduced accumulation of amino acids may be beneficial to plant growth and low N tolerance (Quan et al., 2016; Liu D. et al., 2020). Valine, leucine, and isoleucine are branched-chain amino acids and are critical for protein synthesis while also providing precursors for a number of secondary metabolites. The expression of these amino acids was highly enriched but downregulated in Bluemoon when exposed to low N, suggesting an adjustment of metabolite synthesis and a potential energy conversion needed to cope with N deficiency. Low N stress resulted in more enriched degradation DEGs and less enriched biosynthetic DEGs for valine, leucine, and isoleucine in Bluemoon (Sun et al., 2021), which might contribute to the downregulation of these amino acids in Bluemoon under low N, compared to that in Balin (Figure 4A). Exogenous application of these three amino acids provided greater shoot density in creeping bentgrass (*Agrostis stolonifera* L.) compared with urea application (Mertz et al., 2019), demonstrating a role of these amino acids on plant growth. Asparagine (Asn) and glutamine (Gln) are critical for N recycling and remobilization at the whole plant level (Hayashi and Chino, 1990). The downregulation of Asn in Bluemoon and downregulation of Gln in Balin indicated reduced synthesis potentially influenced by a short supply of carbon skeleton from the TCA cycle or enhanced catabolism.

Alterations of other metabolites could also play a role in illustrating differential N responses of Kentucky bluegrass cultivars. GABA is a ubiquitous four-carbon, non-protein amino acid. Carbon skeletons enter the TCA cycle through the GABA shunt, a bypass of the TCA cycle, whereby GABA can function as a nitrogen storage metabolite in plants and play an important role in C-N balance during stress responses (Kinnersley and Turano, 2000; He et al., 2021). The downregulation of GABA in Balin was consistent with lower levels of glutamic acid and glutamine, while the unchanged GABA in Bluemoon may ensure efficient N use, which could be essential for maintaining C-N balance. Exogenous GABA application increased the non-structural carbohydrates, TCA intermediates, and antioxidant enzymes in poplar (*Populus* spp.), supporting that GABA affected C:N and plant growth by reducing energy costs under low N conditions (Chen et al., 2020).

Melatonin (*N*-acetyl-5-methoxytryptamine) is found to delay senescence of plants and improves plant growth (Hernández-Ruiz et al., 2005; Krystyna et al., 2009). Exogenous application of melatonin enhanced plant tolerance to abiotic stresses such as drought, salinity, and temperatures (Tal et al., 2011; Shi et al., 2015; Wei et al., 2015). In this study, a significant upregulation of 5-methoxytryptamine was found in Bluemoon under LN, but no changes of this metabolite were noted in Balin (Supplementary Tables 1, 2). The results suggested a role of melatonin in promoting low N tolerance in Kentucky bluegrass. In addition, a few metabolites that were differentially expressed between the two N treatments also seemed interesting. For example, cholecalciferol, also known as one form of vitamin D3, was upregulated in both cultivars under LN, with a slightly higher fold change in Balin (Supplementary Tables 1, 2). Previous studies demonstrated that vitamin D3 sterols might increase DNA synthesis and activate the Ca^2+^ messenger system, thereby enhancing plant root growth (Phythoud et al., 1986; Vega et al., 1988). The upregulation of vitamin D3 suggested a role of this compound in mediating plant responses to N stress in Kentucky bluegrass. However, the mechanisms of its regulating plant stress responses deserve further investigations. Polyamines are ubiquitously present in all plant tissues. A high cellular level of polyamines is associated with plant tolerance to various abiotic stresses (Handa et al., 2018). Exogenous spermine application can partially counteract the damage caused by nitrogen deficiency in a medicinal plant (Rubio-Rodríguez et al., 2019). Spermidine, one of the major polyamines, was upregulated in both cultivars under LN, with a higher fold change observed in Balin (Supplementary Tables 1, 2). It appeared that N deficient condition stimulated spermidine biosynthesis, especially in the N-sensitive cultivar of Kentucky bluegrass. This may indicate a need for metabolic adaptation to low N supply.

**FIGURE 6:**
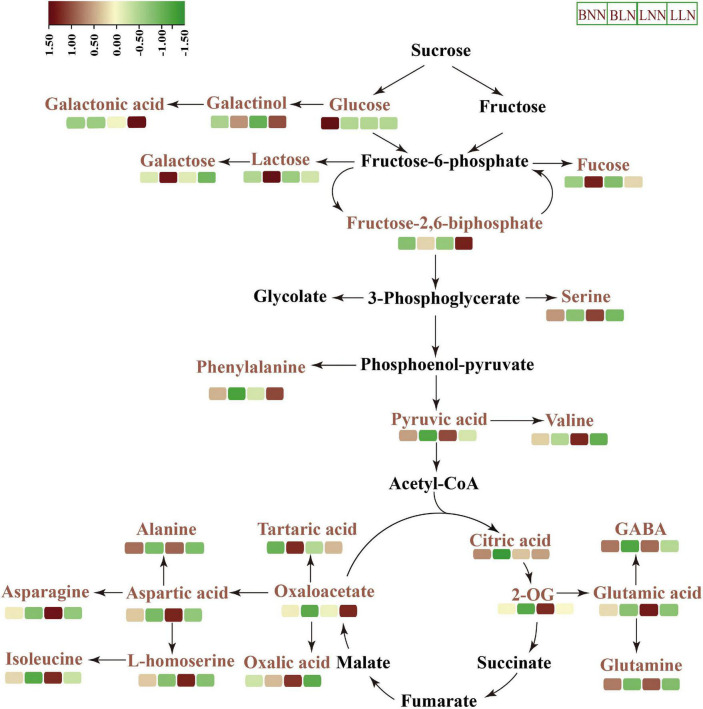
Schematic representation of key metabolites in carbon–nitrogen metabolic pathways under normal nitrogen (NN) and low nitrogen (LN) treatments. BNN, Balin under NN; BLN, Balin under LN; LNN, Bluemoon under NN; LLN, Bluemoon under LN.

Collectively, two Kentucky bluegrass cultivars had similar trends in some metabolites but differed in others in response to LN (Figures 4, 6). In general, the tolerant Bluemoon maintained a higher level of the TCA cycle and had more downregulated amino acids than the sensitive Balin, while changes of more organic acids occurred to Balin. This may lead to higher energy production and low energy costs in Bluemoon, contributing to its greater low N tolerance. However, the results also reflected that some metabolite changes included by low-N stress were cultivar or genotype-specific (Figure 6). The interconnections of C-N metabolism of both cultivars illustrated their unique way in responding or coping with the inhibition of growth and physiology caused by N deficiency. Further in-depth knowledge of these interconnections could help better understand plant N metabolism in perennial grass species.

## Data Availability Statement

The original contributions presented in the study are included in the article/Supplementary Material, further inquiries can be directed to the corresponding authors.

## Author Contributions

XS performed the data analysis and wrote the manuscript. ZG, ZS, LQ, LD, LX, RY, and WD helped in data interpretation. YJ contributed to data interpretation and manuscript writing. FX and YC designed and supervised the experiment. All authors approved the publication of the manuscript.

## Conflict of Interest

The authors declare that the research was conducted in the absence of any commercial or financial relationships that could be construed as a potential conflict of interest.

## Publisher’s Note

All claims expressed in this article are solely those of the authors and do not necessarily represent those of their affiliated organizations, or those of the publisher, the editors and the reviewers. Any product that may be evaluated in this article, or claim that may be made by its manufacturer, is not guaranteed or endorsed by the publisher.
